# Redefine the Role of Proton Beam Therapy for the Locally-Advanced Non-Small Cell Lung Cancer Assisting the Reduction of Acute Hematologic Toxicity

**DOI:** 10.3389/fonc.2022.812031

**Published:** 2022-06-30

**Authors:** Xi Cao, Peilin Liu, Xian-shu Gao, Shiyu Shang, Jiayu Liu, Zishen Wang, Mengmeng Su, Xuanfeng Ding

**Affiliations:** ^1^Department of Radiation Oncology, Peking University First Hospital, Beijing, China; ^2^Institute of Medical Technology, Peking University Health Science Center, Beijing, China; ^3^Department of Oncology, Hebei North University, Zhangjiakou, China; ^4^Department of Radiation Oncology, Hebei Yizhou Tumor Hospital, Zhuozhou, China; ^5^Department of Radiation Oncology, Peking University International Hospital, Beijing, China; ^6^Department of Radiation Oncology, Beaumont Health, Proton Beam Therapy Center, Royal Oak, MI, United States

**Keywords:** NSCLC, NTCP, hematologic toxicity, IMPT, VMAT

## Abstract

**Purpose:**

To investigate the potential clinical benefit of utilizing intensity-modulated proton therapy (IMPT) to reduce acute hematologic toxicity for locally advanced non-small cell lung cancer (LA-NSCLC) patients and explore the feasibility of a model-based patient selection approach *via* the normal tissue complication probability (NTCP).

**Methods:**

Twenty patients with LA-NSCLC were retrospectively selected. Volumetric modulated arc photon therapy (VMAT) and IMPT plans were generated with a prescription dose of 60 Gy in 30 fractions. A wide range of cases with varied tumor size, location, stations of metastatic lymph nodes were selected to represent the general cancer group. Contouring and treatment planning followed RTOG-1308 protocol. Doses to thoracic vertebral bodies (TVB) and other organ at risks were compared. Risk of grade ≥ 3 acute hematologic toxicity (HT3+) were calculated based on the NTCP model, and patients with a reduction on NTCP of HT3+ from VMAT to IMPT (△NTCP_HT3+) ≥ 10% were considered to ‘significantly benefit from proton therapy.’

**Results:**

Compared to VMAT, IMPT significantly reduced the dose to the TVB, the lung, the heart, the esophagus and the spinal cord. Tumor distance to TVB was significantly associated with △NTCP _HT3+ ≥ 10%. For the patients with tumor distance ≤ 0.7 cm to TVB, the absolute reduction of dose (mean, V30 and V40) to TVB was significantly lower than that in patients with tumor distance > 0.7 cm.

**Conclusion:**

IMPT decreased the probability of HT3+ compared to VMAT by reducing the dose to the TVB in LA-NSCLC patients. Patients with tumor distance to TVB less than 0.7 cm are likely to benefit most from proton over photon therapy.

## Introduction

Lung cancer is the most commonly diagnosed cancer and the leading cause of cancer-related death, responsible for 18% of cancer mortalities, in which non-small cell lung cancer (NSCLC) accounts for most of all new diagnoses (1). Approximately three-quarters of lung cancers are locally advanced or advanced at the time of diagnosis, requiring multi-disciplinary management (2). For locally advanced NSCLC (LA-NSCLC), concurrent chemoradiotherapy (CCRT) is the first-line treatment recommended by the guidelines (3, 4). However, it is reported that over 40% of patients suffered from ≥ grade 3 acute hematologic toxicity (HT3+) during CCRT with photon therapy (3, 5). Severe hematologic toxicity (HT) can result in disruption of chemotherapy or reduction on the prescription dose, disabling patients from completing standard treatment (6, 7). Besides, several hematologic events have been proven to be associated with the development of other treatment-related complications and poor clinical outcomes. For example, previous studies have shown that neutropenia was strongly associated with RT-induced dysphagia/esophagitis (8), and grade ≥ 3 anemia and increased neutrophil-to-lymphocyte ratio were associated with compromised survival (9, 10).

Bone marrow cells are highly radiosensitive. Uncompensated injury to hematopoiesis with the massive destruction of both mature cells and precursors can occur when large fields of bone marrow are irradiated, which later manifests as a rapid decline in peripheral blood cells (11). In fact, nearly 30% of bone marrow is located in the thorax region (12). Deek’s work has proven that hematologic toxicity risk was associated with the volume of the thoracic vertebral bodies (TVB) receiving low-dose radiation (13). Based on their work, Barney et al. have established the Lyman–Kutcher–Burman (LKB) model for acute HT in patients receiving CCRT for lung cancer (7), further establishing the link between radiation dose and the occurrence of HT3+.

Proton beam therapy offers a significant dosimetric advantage over conventional photon radiotherapy in terms of organs at risk (OARs) sparing utilizing its unique physical properties of the Bragg peak (14, 15). Numerous clinical studies have demonstrated the superiority in reducing normal tissue toxicity, such as cardiopulmonary toxicity, esophagitis, and dermatitis, in NSCLC (16–18). Although Sejpal’s work demonstrated no difference in HT risk between 3D conformal proton therapy (3DCPT) and other forms of photon therapy (19). Newer proton therapy technique, such as pencil beam therapy, offers a significant dosimetric advantage and clinical outcome over 3DCPT (20–22). Thus, decreasing the risk of HT *via* pencil beam scanning technique may be promising.

We hypothesize that intensity-modulated proton therapy (IMPT) could effectively reduce the dose to TVB without compromising the dose to the target, potentially reducing the incidence of acute HT3+. Based on the patient geometry and normal tissue complication probability (NTCP) model results, this study identified a subgroup of LA-NSCLC patients who may benefit most from acute HT reduction using IMPT. To our best of knowledge, this is the first investigation evaluating the incidence of HT in LA-NSCLC patients in comparison with photon and pencil beam therapy. Such work could guide future model-based optimal treatment modality selection in LA-NSCLC patients with CCRT.

## Method

### Patient, Target Volume, and OAR Definition

A total of twenty patients with LA-NSCLC were retrospectively selected in this study, providing a variety of patient geometry such as different tumor sizes, locations, and distances to vertebrae bodies (**Table 1**). All these cases underwent photon radiotherapy at our institution, and the Institutional Review Board approved using the patient data. All patients received Four-dimensional (4D) CT simulation for planning and contouring. Gross Tumor Volumes (GTVs) (the primary tumor and positive lymph nodes) were contoured on all 4D-CT phases and combined into Internal GTV (IGTV). Clinical Tumor Volume (CTV) was generated with an 8mm expansion of GTVs and edited according to anatomic boundaries. 5mm symmetrical expansion of CTV formed Planning Tumor Volume (PTV) for VMAT plans. OAR structures, including left lung, right lung, bilateral lungs, heart, esophagus, and sometimes trachea if the tumor was centrally located, were delineated based on RTOG-1308 (23). TVB from T1 to T10 was delineated as the surrogate for thoracic bone marrow (7).

**Table 1:** Disease characteristics of the twenty proxy cases.

Patient (No.)	GTV (cc)	Staging (AJCC 8th)	Primary tumor location	IASLC Lymph node stations	Distance to TVB (cm)
1	181.3	IIIB (T4N2M0)	RUL	4R,10R	1.61
2	495.5	IIIC (T4N3M0)	RUL	1R	0.00
3	130.0	IIIC (T3N3M0)	LUL	4L,4R,5,6,7	0.73
4	89.8	IIIB (T4N2M0)	RUL	4R,7	1.98
5	36.6	IIIB (T2N3M0)	RUL	1R,2R,4R,10R	0.26
6	72.8	IIIC (T3N3M0)	LUL	1L	1.03
7	234.2	IIIB (T4N2M0)	RUL & RML	4R	1.10
8	236.0	IIIC (T4N3M0)	RUL	2R, 4L, 4R	0.00
9	225.3	IIIA (T4N0M0)	LUL & LLL	/	2.10
10	255.1	IIIC (T4N3M0)	RUL	1R,1L,2L,4R,4L,7,10R	0.21
11	390.4	IIIC (T4N3M0)	RUL	1R	0.00
12	22.8	IIIA (T1N2M0)	RLL	4R,5	1.40
13	95.4	IIIA (T2N2M0)	RUL	4R,7,10R	0.64
14	58.2	IIB (T2N1M0)	LUL & LLL	10L	1.30
15	26.4	IIIB (T2N3M0)	LUL	2R,4L,4R	1.39
16	38.6	IIIB (T2N3M0)	RLL	4L	0.00
17	157.7	IIIA (T3N1M0)	LLL	10L	1.06
18	23.9	IIIC (T3N3M0)	LUL	4R,10	0.53
19	186.4	IIIA (T3N1M0)	RUL	10R	0.78
20	176.3	IIIC (T2N3M0)	LLL	4L,4R,7,10L	0.00

GTV, gross tumor volume (includes primary and nodal spread); TVB, thoracic vertebral bodies; HT3+, grade ≥ 3 hematologic toxicity.

Location definitions: RUL, right upper lobe; LUL, left upper lobe; RML, right middle lobe; RLL, right lower lobe; LLL, left lower lobe.

AJCC, American Joint Committee on Cancer.

IASLC, The International Association for the Study of Lung Cancer.

### Treatment Planning

Both VMAT and robust-optimized IMPT (RO-IMPT) plans were generated using Raystation version 7.0 (RaySearch Laboratory AB, Stockholm). VMAT plans were created with two or four arcs using a Varian linear accelerator (Trilogy, Varian medical system, Inc., Palo Alto, California). 95% volume of the PTV was requested to receive the prescription dose. Two or three fields single-field optimization (SFO) planning approach was used in RO-IMPT plans based on the beam model from an IBA ProteusPLUS^®^ system. To satisfy the prescription dose to clinical target, a CTV-based robust optimization was used with parameters ± 3mm for x, y, z directions and a range uncertainty of 3.5% (24). The Monte Carlo dose calculation was used with the value 1.1 for the generic relative biological effectiveness (RBE) (25). Both prescription dose of the photon and proton was 60 GyE in 30 fx for the target. Besides, the constraints of OARs were based on RTOG-1308 for both photon and proton plans.

### Planning Robustness and Quality Evaluation

To evaluate the robustness of the IMPT plan quality, 21 worst scenarios were created for all RO-IMPT plan with 3 mm isocenter shift in the direction of anterior-posterior (A-P), superior-inferior (S-I), and right-left (R-L) with + 3.5%, 0, and −3.5% proton beam range uncertainties according to the nominal proton plan (24, 26). The criteria set of robustness are as follows: for the CTV: D90 ≥ 95% for the worst scenarios and D95 ≥ 95% for all scenarios. The conformal index (CI) of CTV was also used to assess the plan quality of both proton and photon plans. CI was defined as:


CI=TVI/TIV,

Where *TVI* represents 95% of the target volume covered by the prescription dose and *TIV* represents the total isodose volume. The closer to 1, the better the conformity of the target. Besides, the detailed evaluation for all OARs was based on the constraints of RTOG 1308 (23).

### Evaluation of NTCP

The incidence of HT3+ was calculated from the LKB-NTCP model shown in the following equations (27):


(1) $$NTCP=\frac{1}{\sqrt{2\pi }}\int_{-\infty }^{t}e^{-\frac{x^{2}}{2}}\;\;dx$$

Where,


(2) $$t=\frac{EUD-TD_{50}}{mTD_{50}}$$

TD_50_ represents the threshold dose with a 50% probability of complication in the corresponding organ; The parameters m and n are described as the slope of the dose-response curve and the volume dependence of the NTCP, respectively. The formula of equivalent uniform dose (EUD) is as follows:


(3) $$EUD={\left(\sum_{i}v_{i}D_{i}^{\frac{1}{n}}\right)}^{n}$$

It means that the volume *v_i_* in the correlated organ has received a uniform dose *D_i_*. Matlab version R2019b (MathWorks Inc., Natick, Massachusetts, USA) was utilized to calculate NTCP values. In this study, the parameters (n, m, and TD_50_) were derived from *Barney*, which were set as 1, 1.79, and 21.4 Gy, individually (7).

### Reduction on NTCP of HT3+ (△NTCP_HT3+)

△NTCP_HT3+ was calculated for each patient. Consistent with previous studies, a NTCP reduction of over 10% was considered as a ‘significant reduction’ (28, 29). In this study, patients with △NTCP_HT3+ ≥ 10% were considered as ‘benefit most from PT in terms of HT3+ mitigation’.

### Statistical Analysis

Statistics analyses were performed using SPSS version 24.0 software (IBM, Armonk, New York). Univariate analysis was done to determine the factors related to △NTCP_HT3+. The odds ratio was calculated to estimate and compare the chance of the event of △NTCP_HT3+ > 10% in the patient subgroups based on the tumor distance to the TVB. Paired t-test and two related sample non-parameter tests (Wilcoxon signed-ranked test) were conducted to compare the dosimetric parameters between IMPT and VMAT plans for the CTV and OARs. Independent-sample t-test and nonparametric tests (Mann-Whitney test) were conducted to compare the absolute dose reductions on OARs. P < 0.05 was considered statistically significant.

## Results

### Disease Characteristics

Details of disease characteristics of the twenty LA-NSCLC patients, including staging, distribution of primary tumor and lymph node stations, and the closest distance of GTV to thoracic vertebral bodies, were summarized in **Table 1**. The majority of patients were stage III (19/20) and had nodal involvement (19/20). Among the 20 patients, one had T4N0 disease (patient #9), and one had nodal positive stage IIB disease (patient #14). Tumor volume ranged from 22.8 - 495.5cc with a median of 143.9 cc. The closest distance of tumor to TVB ranged from 0-2.1cm with a median of 0.8 cm.

### Planning Quality Evaluation

**Figure 1:**
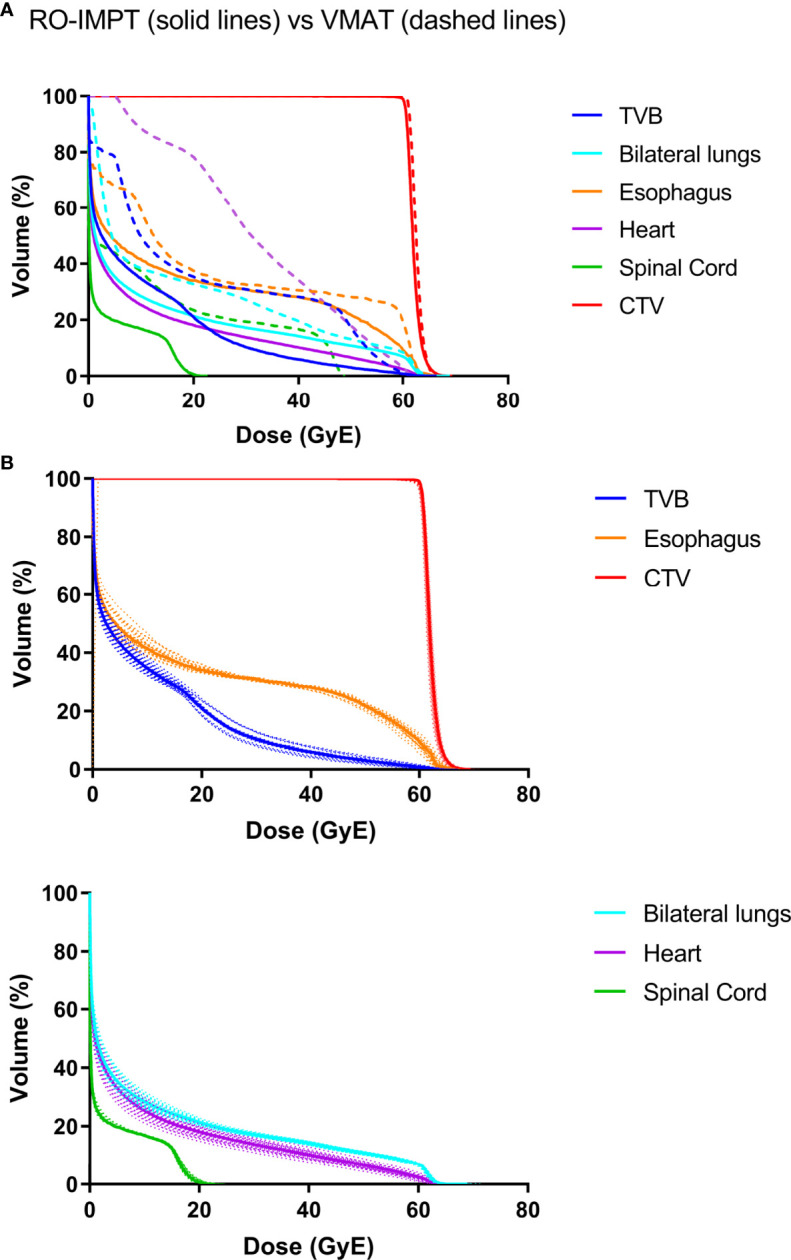
**(A)** A representative dose volume histogram (DVH) of patient #20. The solid line is RO-IMPT, and the dashed line is VMAT. **(B)** DVHs perturbation of the 21 worst scenarios of RO-IMPT. TVB, thoracic vertebral bodies.

All VMAT and RO-IMPT plans reached the prescription dose of the target. RO-IMPT provided a better CI (median: 0.57) compared to VMAT (median: 0.47) (p < 0.001). The dosimetric volume histogram of target and OARs of VMAT were represented in **Figure 1A** (patient #20). RO-IMPT plan provided a robust target coverage (D95 > 95%) in all 21 worst-case scenarios for each patient. **Figure 1B** showed an example of dose volume histogram (DVH) perturbation of the 21 worst scenarios of the same patient (#20).

### NTCP of Grade ≥ 3 Acute Hematologic Toxicity (NTCP_HT3+)

**Figure 2:**
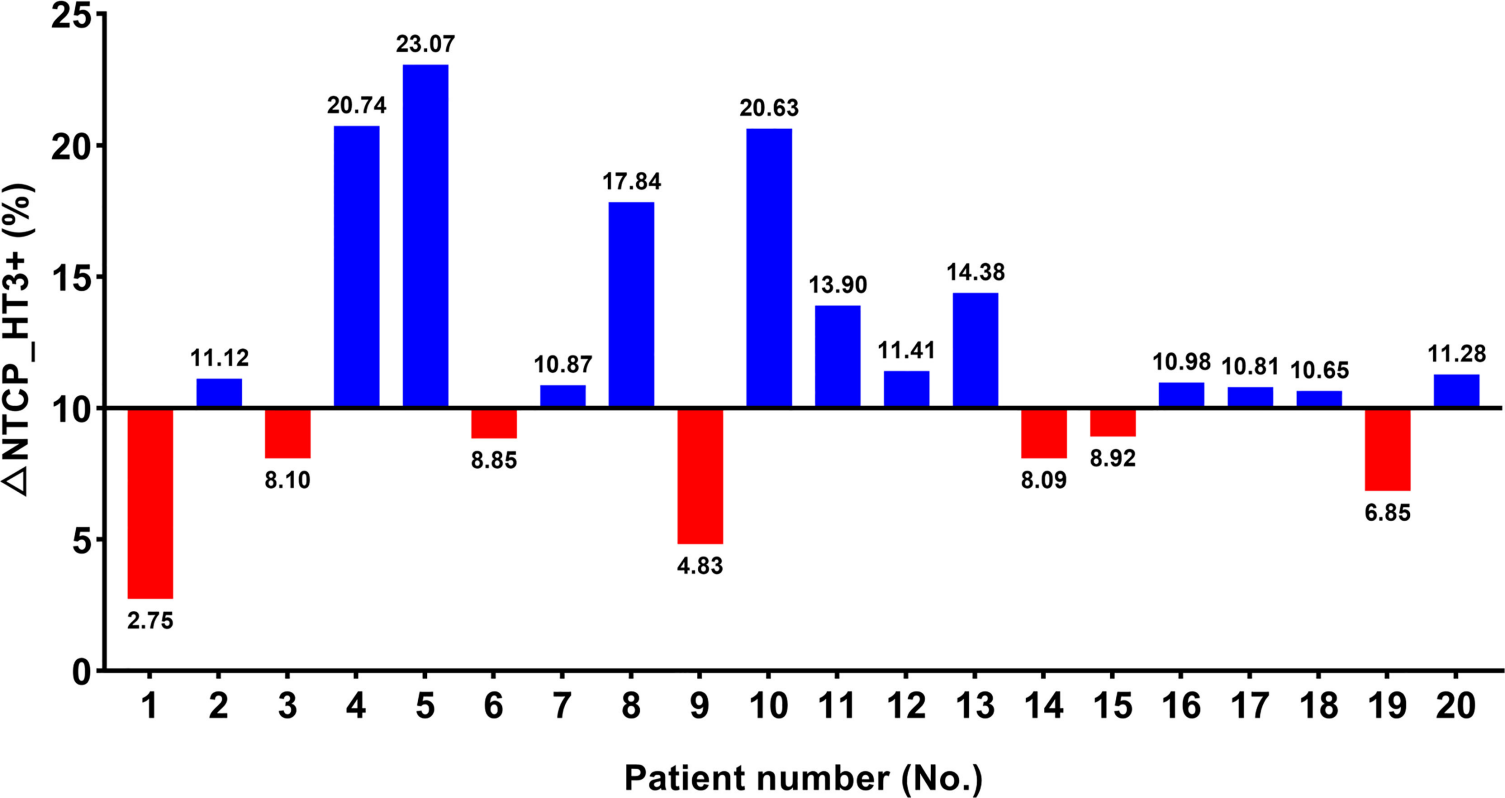
Possibility of grade ≥ 3 hematologic toxicities (HT3+) in twenty patients. △NTCP_HT3+ ≥ 10% were considered as ‘benefit most from IMPT in terms of HT3+ mitigation’.

NTCP_HT3+ was calculated for twenty cases and listed in **Table A1**. The study found that NTCP_HT3+ with IMPT was significantly lower compared to VMAT (36.69 ± 6.07 vs. 47.86 ± 8.88, p < 0.001). Seven patients presented with △NTCP_HT3+ < 10%, and the other thirteen patients presented with △NTCP_HT3+ ≥ 10% (**Figure 2** and **Table A1**). Univariate analysis showed that the closest distance of the tumor to TVB was significantly different between the subgroup with △NTCP_HT3+ < 10% and the subgroup with △NTCP_HT3+ ≥ 10% (1.28 ± 0.48 vs. 0.55 ± 0.65, p=0.024), while other characteristics were not (**Table 2**).

**Table 2:** Univariate analysis of variables associated with △NTCP_HT3+.

Parameters	△NTCP			t/χ2 test
	<10% (N = 7)		≥10% (N = 13)	P value
Distance to TVB (cm)*	1.28 ± 0.48		0.55 ± 0.65	0.024**
Volume (cc)				
GTV	125.77 ± 75.20		173.25 ± 147.30	0.439
GTVp*	112.26 ± 78.36		141.38 ± 152.71	0.877
GTVnd*	13.51 ± 21.73		31.87 ± 61.61	0.588
Disease location				
TVB level_top	2.57 ± 1.99		2.69 ± 2.39	0.911
TVB level_bottom*	6.29 ± 1.80		7.69 ± 1.32	0.081
Average of TVB level	4.43 ± 1.84		5.19 ± 1.70	0.364

GTV, gross tumor volume (includes primary and nodal spread); GTVp, gross tumor volume of primary site; GTVnd, gross tumor volume of metastatic lymph nodes; TVB, thoracic vertebral bodies; TVB level_top, the highest thoracic vertebra level that the disease across; TVB level_bottom, the lowest thoracic vertebra level that the disease across; Number of V, numbers of vertebrae of the tumor across; average of TVB level, the average of TVB level_top and TVB level_bottom.

*Non-normal distribution parameters were analyzed with nonparametric tests (Mann-Whitney test); Otherwise, normal distribution parameters were analyzed with independent-sample t test.

**p < 0.05; otherwise, p ≥ 0.05.

A Chi-square test was performed to determine the association between △NTCP _HT3+≥ 10% and different distance values between 0.1 cm to 2.0 cm with an interval of 0.1cm (**Table A2**). Results show that distance < 0.3 cm (OR 2.167, CI 1.204-3.898), distance < 0.4 cm (OR 2.167, CI 1.204-3.898), distance < 0.5 cm (OR 2.167, CI 1.204-3.898), distance < 0.6 cm (OR 2.600, CI 1.307-5.171), and distance < 0.7 cm (OR 3.250, CI 1.438-7.345) were significantly associated with △NTCP _HT3+≥ 10%. Among them, the condition ‘distance < 0.7 cm’ showed the best sensitivity and specificity to predict a result of △NTCP _HT3+≥ 10%. In this cohort, the sensitivity and specificity of the condition ‘distance < 0.7 cm’ were 69.2% and 100.0%, respectively.

### Dosimetric Comparison Between IMPT vs. VMAT

**Figure 3:**
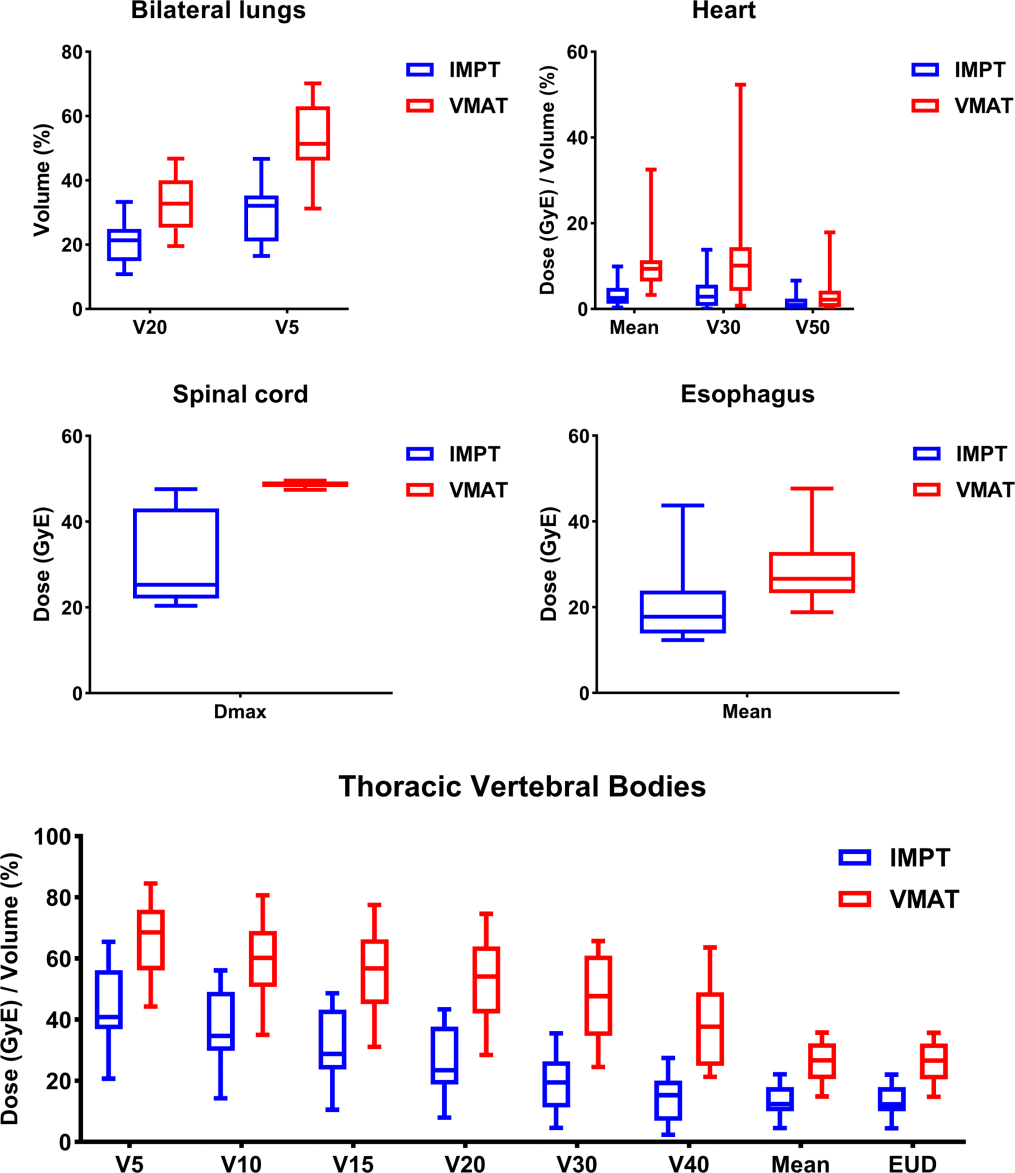
Dosimetric results of the subgroup patients with tumor distance ≤ 0.7cm to thoracic vertebral bodies.

For the whole cohort, IMPT significantly reduced the dose to TVB and other OARs (e.g., lungs, esophagus, heart, spinal cord) compared to VMAT (**Table A3**). The reduction of maximal dose, mean dose, and the volume of OARs receiving dose ≥ x Gy (Vx) was calculated for each patient. Patients were divided into two groups according to their closest tumor distance to TVB (‘≤ 0.7cm’ group with distance ≤ 0.7cm and ‘> 0.7cm’ group with distance > 0.7cm). A summary of the dosimetric metrics of the absolute reduction from the subgroups was presented in **Table 3**. Data of the absolute reduction of ‘≤ 0.7cm’ subgroup and ‘> 0.7cm’ subgroup were displayed in **Figure 3** and **Figure A2**, respectively. The absolute dose reduction of V30, V40, and mean dose of TVB were significantly higher in ‘≤ 0.7cm’ subgroup compared to ‘> 0.7cm’ subgroup (p < 0.05), so as to the absolute reduction of lung V20. This difference was not observed in other OARs such as the heart, esophagus, and spinal cord.

**Table 3:** Comparison of absolute reduction on dosimetric parameters of organs at risk.

Parameters	Absolute reduction		P value
	Distance ≤ 0.7cm	Distance > 0.7cm	
TVB			
V5 (%)	22.47 ± 12.09	32.32 ± 11.01	0.073
V10 (%)*	22.85 ± 11.25	30.06 ± 11.38	0.095
V15 (%)*	24.84 ± 12.11	26.99 ± 13.47	0.370
V20 (%)*	26.77 ± 11.84	24.18 ± 13.40	0.766
V30 (%)*	28.01 ± 10.02	16.40 ± 14.89	0.006**
V40 (%)*	24.31 ± 9.24	8.18 ± 9.35	0.002**
Dmean (GyE)*	12.77 ± 4.38	9.75 ± 4.38	0.020**
Lung			
V5 (%)	22.91 ± 12.78	21.43 ± 5.88	0.735
V20 (%)	11.77 ± 4.72	7.16 ± 3.65	0.024**
Heart			
V30 (%)*	9.51 ± 11.38	6.92 ± 7.32	0.656
V50 (%)*	2.21 ± 3.54	5.43 ± 12.93	0.710
Dmean (GyE)*	7.54 ± 5.86	5.49 ± 4.31	0.503
Esophagus			
Dmean (GyE)	8.61 ± 5.20	7.65 ± 5.22	0.688
Spinal cord			
Dmax (GyE)*	17.89 ± 10.59	19.88 ± 8.78	0.656

TVB, thoracic vertebral bodies; Dmean, mean dose; Dmax, maximal dose.

*Non-normal distribution parameters were analyzed with nonparametric tests (Mann-Whitney test); Otherwise, normal distribution parameters were analyzed with independent-sample t test.

**p < 0.05; otherwise, p ≥ 0.05.

## Discussion

The high incidence of HT during CCRT in lung cancer treatment has long been a challenge, as studies have shown severe HT is the prognostic factor for poor patient outcomes (8–10). Through a comprehensive dosimetric comparison of IMPT and VMAT, this study investigated the potential clinical hematologic benefits of utilizing IMPT in the treatment of LA-NSCLC. The result shows that the average probability of HT3+ in the VMAT group is 48%, which is consistent with past clinical reports from photon therapy (3, 5, 7). The results showed that an average of 11% of the probability of HT3+ was reduced *via* IMPT from VMAT (**Table A1**). Notably, the percentage of △NTCP_HT3+ over 10% was significantly higher in the subgroup with the property of closest tumor distance < 0.7 cm to vertebral bodies (OR 2.75, 95% CI 1.26 - 6.02). Additionally, the dose to TVB was reduced across all DVH parameters (TVB V5-40 and Dmean). TVB V30 and Dmean showed a more significant absolute reduction in the subgroup with close tumor distance to TVB. A dosimetric study has revealed that proton therapy can reduce 30% volume in the bone marrow receiving a dose of 10 Gy (30). The higher risk of hematologic toxicity during CCRT has been linked to the larger volume of bone marrow receiving a low-to-medium dose range, and several studies have demonstrated the association between HT3+ and DVH parameters (TVB V20, TVB V30, and Dmean) (7, 13, 31). These findings support our hypothesis that IMPT could reduce the probability of HT3+ *via* reducing the dose to TVB, and this effect may be enhanced in those who have tumors close to TVB.

Preliminary results from studies with a small sample size have shown a low HT3+ risk of CCRT with proton therapy (32, 33). Recent closed single-arm clinical trials NCT00881712, NCT00495170, NCT01076231 reached similar conclusions (34–36). However, the comparative study led by Sejpal reported no difference in hematologic toxicities between proton therapy and photon therapy (19). This report has several limitations in the present context. On the one hand, a substantial difference in pretreatment status and treatment delivery existed in this non-randomized study. More cases of recurrent disease and older patients were included in the proton group, and the median prescription dose was 11 GyE higher in the proton group than the photon group. Basically, proton therapy achieved a comparable HT result with possibly worse patient bone marrow reserve and higher prescription dose. On the other hand, this study compares the 3DCPT with other forms of photon therapy. An advanced technology, pencil beam proton therapy, can deliver better dose conformity to the target compared to the passive-scattering technique. Multiple studies have confirmed that the advancement of technology is associated with decreased toxicity and comparable or even better survival (21, 37, 38). As for pencil beam therapy, studies have confirmed the dosimetric advantage of IMPT over PSPT (18, 20, 39). A newly published clinical trial also revealed the decreased risk of cardiopulmonary toxicity and a tendency of improved survival with IMPT than PSPT (18). Thus, with the advanced proton technology, IMPT, a benefit in HT is expected in lung cancer patients, at least in the selected patient group.

Although PT shows dosimetric superiority over photon therapy in the management of LA-NSCLC, it is unrealistic to simply replace photon with proton for all patients due to the high cost and limited availability (14). The result from the study indicated that the potential clinical benefits of HT3+ reduction depended on the patient geometry, e.g., correlation with the distance between the TVB and targets. This model-based approach from the study could provide practical and clinical guidance in selecting proton beam therapy for LA-NSCLC patients to reduce the probability of HT3+. Although our study did not provide clinical data to validate the model-based approach, it can serve as a good starting point for future studies. To our knowledge, there are no clinical studies that include HT3+ as endpoints in proton therapy for NSCLC at present. We hope that such clinical studies will emerge in the future to verify our findings. After validation, for those with a high risk of NTCP_HT3+, regardless of treatment modalities, medications such as white blood cell growth factors can be used prophylactically to guarantee the completion of the whole treatment, decrease hospitalization, and reduce hospitalization economic burden (40–42).

Based on the study results, the following research direction might be worth pursuing for LA-NSCLC patient populations. First, Spot-scanning Proton Arc therapy (SPArc), an advanced IMPT delivery technique, could provide potential clinical benefits in dose conformity, mitigation of interplay effect utilizing the additional degrees of optimization freedom (43, 44). Utilization of SPArc may further increase the role of proton therapy in HT reduction. Second, the radiation to the heart, the great vessels, and lymphoid organs such as bone marrow also play a role in immunosuppression (9, 45, 46). With the PACIFIC study announcing the coming of the era of immune-oncology in the treatment of LA-NSCLC, lymphocyte preservation during CCRT has been drawing significant interest (47–49). Developing a model to predict the possibility of lymphopenia will also be a good contribution in this field.

The limitation of our study is that Barney’s model is based on photon therapy. Whether or not it is effective for proton has no verdict. Nevertheless, it is still a plausible way to assess HT and quantify the difference between treatment modalities (50). This study follows the current clinical planning practice that prioritize the OARs sparing in lung, heart and spinal cord. The TVB sparing was not put in the plan optimization. At present, it’s challenging to find a specific constraint for TVB because the extra sparing in bone marrow could dump the dose somewhere else, resulting in a higher dose in other OARs that could cause severe complication such as radiation pneumonitis and radiation-induced myocardial damage. And the balance between the protection of bone marrow and other OARs will definitely be a great research direction in the future.

Besides, other clinical factors, such as age, sex, and physical status, may also affect the bone marrow reserve and hematopoiesis, which were not included in the current model. Finally, recent researches have found that with different positions of the pathway of the proton beam, RBE may not be a consistent 1.1. For instance, the value of RBE has risen to 1.35 at the distal edge, which is possible to influence the clinical benefit of IMPT (51). To address this challenge, the linear energy transfer optimization algorithm or variable RBE model could be implemented clinically (52).

## Conclusions

The present study suggested that IMPT could effectively reduce HT3+ compared to VMAT by decreasing the dose to the TVB in LA-NSCLC patients with CCRT. The most potential clinical benefit was found in patients with tumor distance ≤ 0.7 cm to TVB. This study could be the cornerstone of model-based patient selection for further clinical trials in LA-NSCLC patients with CCRT.

## Data Availability Statement

The raw data supporting the conclusions of this article will be made available by the authors, without undue reservation.

## Ethics Statement

The studies involving human participants were reviewed and approved by Peking University First Hospital Ethics Committee. The patients/participants provided their written informed consent to participate in this study.

## Author Contributions

Study conception and design: X-SG, XD, XC, and PL; Data acquisition: XC, PL, SS, JL, ZW, and MS; Statistical analysis: XC and PL; Drafting of manuscript: XC and PL; Critical editorial and writing contributions: X-SG and XD. All authors have read and agreed to the published version of the manuscript.

## Funding

We are grateful to the funding of China International Medical Foundation (Grant Number: 2019-N-11-07).

## Conflict of Interest

The authors declare that the research was conducted in the absence of any commercial or financial relationships that could be construed as a potential conflict of interest.

## Publisher’s Note

All claims expressed in this article are solely those of the authors and do not necessarily represent those of their affiliated organizations, or those of the publisher, the editors and the reviewers. Any product that may be evaluated in this article, or claim that may be made by its manufacturer, is not guaranteed or endorsed by the publisher.
