# Supplementary material for: Redefine the Role of Proton Beam Therapy for the Locally-Advanced Non-Small Cell Lung Cancer Assisting the Reduction of Acute Hematologic Toxicity
Source: Front Oncol. 2022 Jun 30;12:812031. doi: 10.3389/fonc.2022.812031 (PMC9280487; doi:10.3389/fonc.2022.812031)
Supplement: Supplementary file 1 [file DataSheet_1.docx]

**Supplementary file**

**Table A1. Probability of HT3+ of the twenty cases.**

| **Patient (No.)** | **Probability of HT3+ (%)** | | |
| --- | --- | --- | --- |
|  | **IMPT** | **VMAT** | **△NTCP** |
| 1 | 32.23 | 41.20 | 8.97 |
| 2 | 38.09 | 62.73 | 34.64 |
| 3 | 35.67 | 43.42 | 7.75 |
| 4 | 33.69 | 55.36 | 21.67 |
| 5 | 43.25 | 55.39 | 12.14 |
| 6 | 31.12 | 39.48 | 8.36 |
| 7 | 33.57 | 44.20 | 10.63 |
| 8 | 47.01 | 59.45 | 12.44 |
| 9 | 30.23 | 34.75 | 4.52 |
| 10 | 50.65 | 64.59 | 13.94 |
| 11 | 40.63 | 52.09 | 11.46 |
| 12 | 35.99 | 47.74 | 11.75 |
| 13 | 38.51 | 48.75 | 10.24 |
| 14 | 29.45 | 37.98 | 8.53 |
| 15 | 30.53 | 38.73 | 8.20 |
| 16 | 45.77 | 57.73 | 11.96 |
| 17 | 31.80 | 42.09 | 10.29 |
| 18 | 32.93 | 43.18 | 10.25 |
| 19 | 34.10 | 39.13 | 5.03 |
| 20 | 38.60 | 49.24 | 10.64 |

*Abbreviations.* HT3+, grade ≥ 3 hematologic toxicity. △NTCP, reduced NTCP from IMPT to VMAT.

**Table A2. The ability to predict an △NTCP > 10% result with different distance cutoff values.**

| **Distance Cutoff Value**  **(cm)** | **△NTCP>10%** | | **△NTCP<10%** | | **χ^2^** | **Test Characteristics** | |
| --- | --- | --- | --- | --- | --- | --- | --- |
|  | **N. (close)** | **N.**  **(far)** | **N. (close)** | **N.**  **(far)** |  | **Sensitivity** | **Specificity** |
| 0.1 | 7 | 0 | 8 | 5 | 0.083 | 38.5% | 100.0% |
| 0.2 | 7 | 0 | 8 | 5 | 0.083 | 38.5% | 100.0% |
| 0.3 | 7 | 0 | 6 | 7 | 0.022** | 53.8% | 100.0% |
| 0.4 | 7 | 0 | 6 | 7 | 0.022** | 53.8% | 100.0% |
| 0.5 | 7 | 0 | 6 | 7 | 0.022** | 53.8% | 100.0% |
| 0.6 | 7 | 0 | 5 | 8 | 0.010** | 61.5% | 100.0% |
| 0.7 | 7 | 0 | 4 | 9 | 0.004** | 69.2% | 100.0% |
| 0.8 | 5 | 2 | 4 | 9 | 0.102 | 69.2% | 71.4% |
| 0.9 | 5 | 2 | 4 | 9 | 0.102 | 69.2% | 71.4% |
| 1.0 | 5 | 2 | 4 | 9 | 0.102 | 69.2% | 71.4% |
| 1.1 | 4 | 3 | 2 | 11 | 0.078 | 84.6% | 57.1% |
| 1.2 | 4 | 3 | 2 | 11 | 0.078 | 84.6% | 57.1% |
| 1.3 | 3 | 4 | 2 | 11 | 0.207 | 84.6% | 42.9% |
| 1.4 | 2 | 5 | 1 | 12 | 0.270 | 92.3% | 28.6% |
| 1.5 | 2 | 5 | 1 | 12 | 0.270 | 92.3% | 28.6% |
| 1.6 | 2 | 5 | 1 | 12 | 0.270 | 92.3% | 28.6% |
| 1.7 | 1 | 6 | 1 | 12 | 0.589 | 92.3% | 14.3% |
| 1.8 | 1 | 6 | 1 | 12 | 0.589 | 92.3% | 14.3% |
| 1.9 | 1 | 6 | 1 | 12 | 0.589 | 92.3% | 14.3% |
| 2.0 | 1 | 6 | 0 | 13 | 0.350 | 100.0% | 14.3% |

*Abbreviations.* N. (close), number of cases whose distance was smaller than or equal to the cutoff value. N. (far), number of cases whose distance was larger than the cutoff value.

** p < 0.05; otherwise p ≥ 0.05.

| **Table A3. Dosimetric comparison between IMPT and VMAT plans for the CTV and OARs. Proton doses were presented in Gy (RBE).** | | | |  |
| --- | --- | --- | --- | --- |
|  | | **IMPT**  **(median and IQR)** | **VMAT**  **(median and IQR)** | **P value** |
| CTV |  |  |  |  |
| V60 (%) | 99.00 (99.00-99.00) | 99.86 (99.70-99.96) |  |  |
| CI | 0.57 (0.45-0.69) | 0.47 (0.41-0.59) | <0.001** |  |
| Bilateral lungs |  |  |  |  |
| V20 (%) | 17.82 (13.55-21.89) | 25.90 (22.49-32.66) | <0.001** |  |
| V5 (%) | 29.02 (20.63-31.83) | 48.40 (44.32-56.02) | <0.001** |  |
| Spinal cord |  |  |  |  |
| Dmax (GyE)* | 22.07 (14.25-31.88) | 47.53 (37.67-49.24) | <0.001** |  |
| Heart |  |  |  |  |
| Mean (GyE)* | 2.27 (0.86-3.70) | 8.78 (3.84-11.16) | <0.001** |  |
| V30 (%)* | 2.24 (0.29-5.03) | 9.41 (1.27-13.76) | <0.001** |  |
| V50 (%)* | 0.48 (0.02-2.45) | 2.38 (0.03-4.50) | 0.001** |  |
| Esophagus |  |  |  |  |
| Mean (GyE) | 12.96 (8.39-18.97) | 22.87 (16.49-28.24) | <0.001** |  |
| TVB |  |  |  |  |
| EUD (GyE) | 7.50 (3.57-11.81) | 17.52 (11.60-26.59) | <0.001** |  |
| Mean (GyE) | 6.56 (3.42-11.86) | 17.59 (11.65-26.66) | <0.001** |  |
| V5 (%) | 30.26 (18.50-40.76) | 56.38 (47.14-71.23) | <0.001** |  |
| V10 (%) | 21.39 (10.84-33.88) | 48.72 (39.75-64.19) | <0.001** |  |
| V15 (%) | 16.16 (7.02-28.21) | 42.10 (34.77-61.48) | <0.001** |  |
| V20 (%) | 11.96 (5.04-22.84) | 36.55 (28.57-58.95) | <0.001** |  |
| V30 (%)* | 6.52 (2.30-17.83) | 29.20 (16.16-49.82) | <0.001** |  |
| V40 (%)* | 3.25 (0.66-13.55) | 21.43 (6.01-36.37) | <0.001** |  |

*Abbreviations.* IMPT, intensity-modulated proton therapy. VMAT, volumetric modulated arc photon therapy. CTV, clinical tumor volume (includes primary and nodal spread). TVB, thoracic vertebral bodies. IQR, inter quartile range.

* Non-normal distribution parameters were analyzed with nonparametric tests (Mann-Whitney test); Otherwise, normal distribution parameters were analyzed with independent-sample t test.

** p < 0.05; otherwise p ≥ 0.05.

**
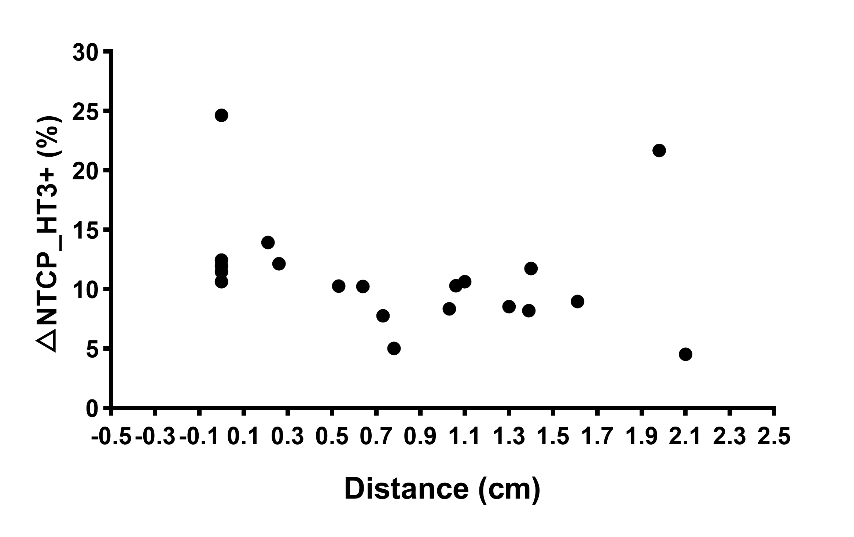
**

**Fig. A1.** Distribution of △NTCP_HT3+ of the twenty patients according to distance.


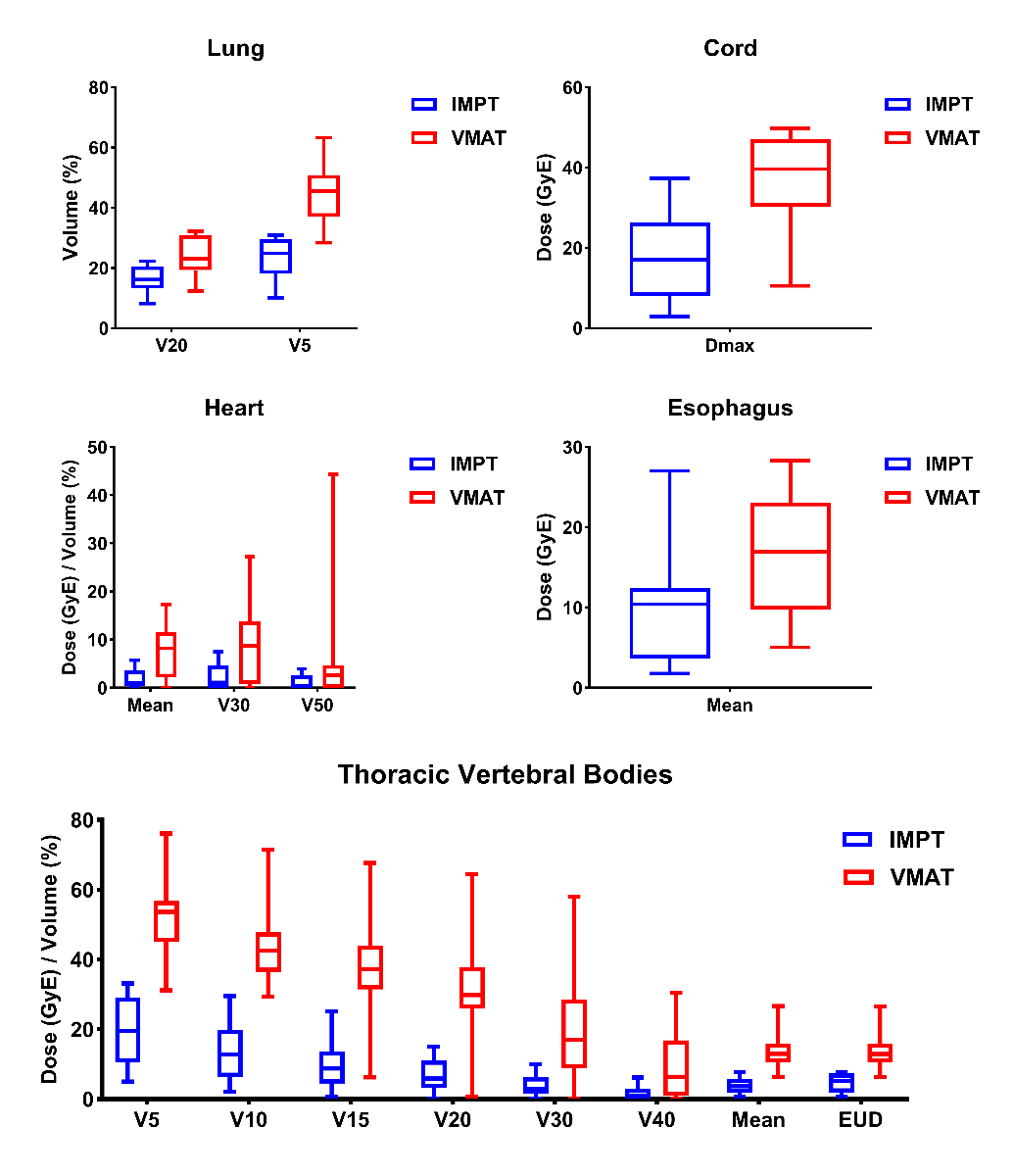


**Fig. A2.** Dosimetric results of the subgroup patients with tumor distance > 0.7cm to thoracic vertebral bodies. *Abbreviations.* EUD, equivalent uniform dose, Vx, correlated volume of being equal or greater than x GyE.
